# Multiple systemic artery to pulmonary vessel fistulas (SAPVFs) completely resected by video-assisted thoracoscopic surgery: a case report

**DOI:** 10.1186/s40792-022-01540-4

**Published:** 2022-09-28

**Authors:** Kyoto Matsudo, Naoki Haratake, Yuki Ono, Mikihiro Kohno, Tomoyoshi Takenaka, Tomoharu Yoshizumi

**Affiliations:** Department of Surgery and Science, Graduate School of Medical Sciences, Kyushu University, 3-1-1 Maidashi, Higashi-Ku, Fukuoka, 812-8582 Japan

**Keywords:** Systemic artery to pulmonary vessel fistula, Multiple, Video-assisted thoracoscopic surgery, Vessel sealing system

## Abstract

**Background:**

Systemic artery to pulmonary vessel fistula (SAPVF) is an uncommon abnormal vascular communication between systemic arteries and the lung parenchyma. It has been reported that the appropriate treatment for SAPVF is embolization or surgical resection. However, in patients such as ours, who have many aberrant vessels or multiple lesions, surgery should be considered as the first-choice treatment.

**Case presentation:**

This case report describes multiple SAPVFs and huge bullae at the apex of the left lung in a 43-year-old man that were resected completely with the video-assisted thoracoscopic surgery (VATS). The patient had an uneventful postoperative recovery without any complications and was discharged 9 days postoperatively. He had heavy smoking history, and the giant bullae and the diffuse emphysematous change were found in the lung. Therefore, the chronic inflammation may have been present in the thoracic cavity, which caused multiple SAPVFs.

**Conclusions:**

We describe the clinical course and management of the patient with multiple SAPVFs who had no obvious history of surgery, trauma, or various inflammatory or infection diseases. VATS should be the first-choice treatment in patients with many abnormal vessels or multiple lesions.

## Introduction

Systemic artery to pulmonary vessel fistula (SAPVF) is an uncommon abnormal vascular communication between systemic arteries and the lung parenchyma [1]. It can be divided into congenital and non-congenital type. Non-congenital SAPVF is caused by pleural adhesions that occur subsequent to cancer, trauma, or infection disease of the lung and pleura. [2]. SAPVF can be treated by embolization or surgical resection. However, in patients with many abnormal vessels or multiple lesions, embolization has been reported to be difficult and the recurrence rate relatively high [2]. In such patients, surgery should be considered as the first-choice treatment.

Here we describe the clinical course and management of the patient with multiple SAPVFs and review the literature on SAPVF.

## Case presentation

**Fig. 1:**
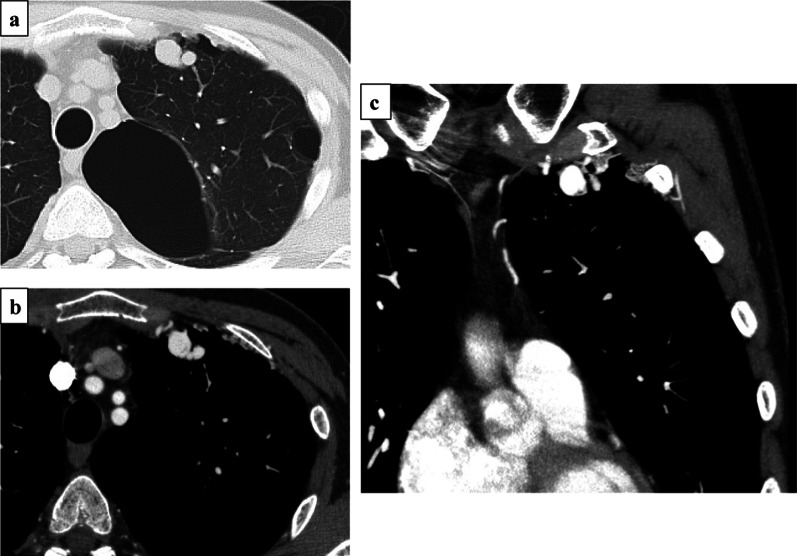
Chest contrast-enhanced computed tomography (CT) scan showed a series of dilated vessels forming a mass-like structure in the anterior segment of left upper lobe. There was a huge bulla at the apex of the lung

**Fig. 2:**
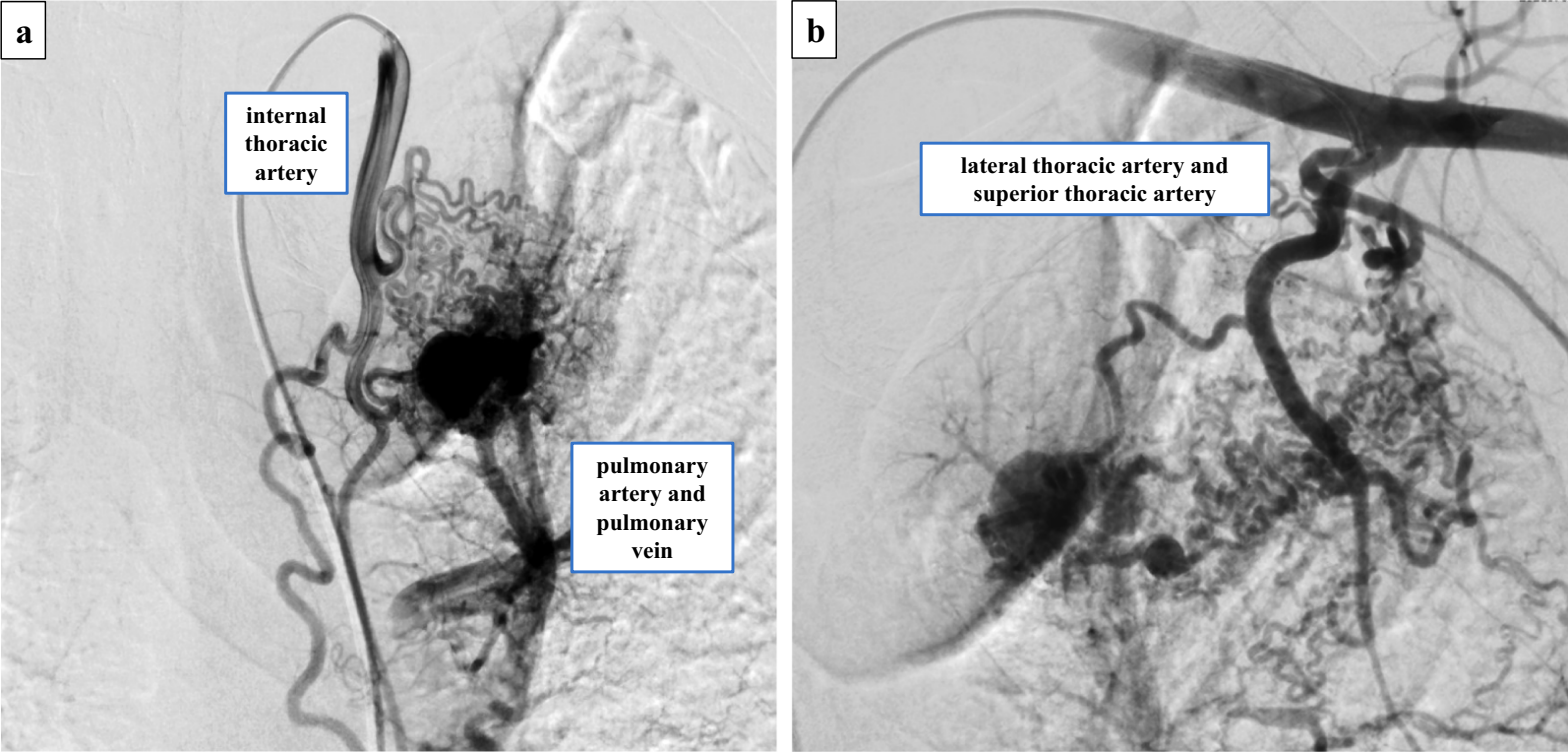
Right femoral arteriography showing three aneurysmal structures approximately 5–15 mm in size, and hypertrophic blood vessels arising from the left internal thoracic artery, lateral thoracic artery, and superior thoracic artery that drain into the both the left upper pulmonary artery and pulmonary vein

A 43-year-old male current smoker presented with abnormal findings on chest computed tomography (CT) that was performed after he presented with backache and dyspnea. The patient had no previous medical history, but he had heavy smoking history. His percutaneous oxygen saturation (SpO2) in room air was 97%. Blood tests revealed no abnormalities. Chest contrast-enhanced CT scan revealed a series of dilated vessels forming a mass-like structure, which was the systemic artery to pulmonary vessel fistula (SAPVF), in the anterior segment of the left upper lobe. There were also huge bullae at the apex of the lung near the SAPVF (Fig. 1). Right femoral arteriography revealed aneurysmal structures approximately 5–15 mm in size and hypertrophic blood vessels that arose from the left internal thoracic artery, lateral thoracic artery, and superior thoracic artery and drained into the both the left upper pulmonary artery and vein (Fig. 2). The cause of dyspnea was thought to be both the SAPVF and huge bullae. Because of the many aberrant blood vessels, embolization was thought to be difficult, and video-assisted thoracoscopic surgery (VATS) was performed.

**Fig. 3:**
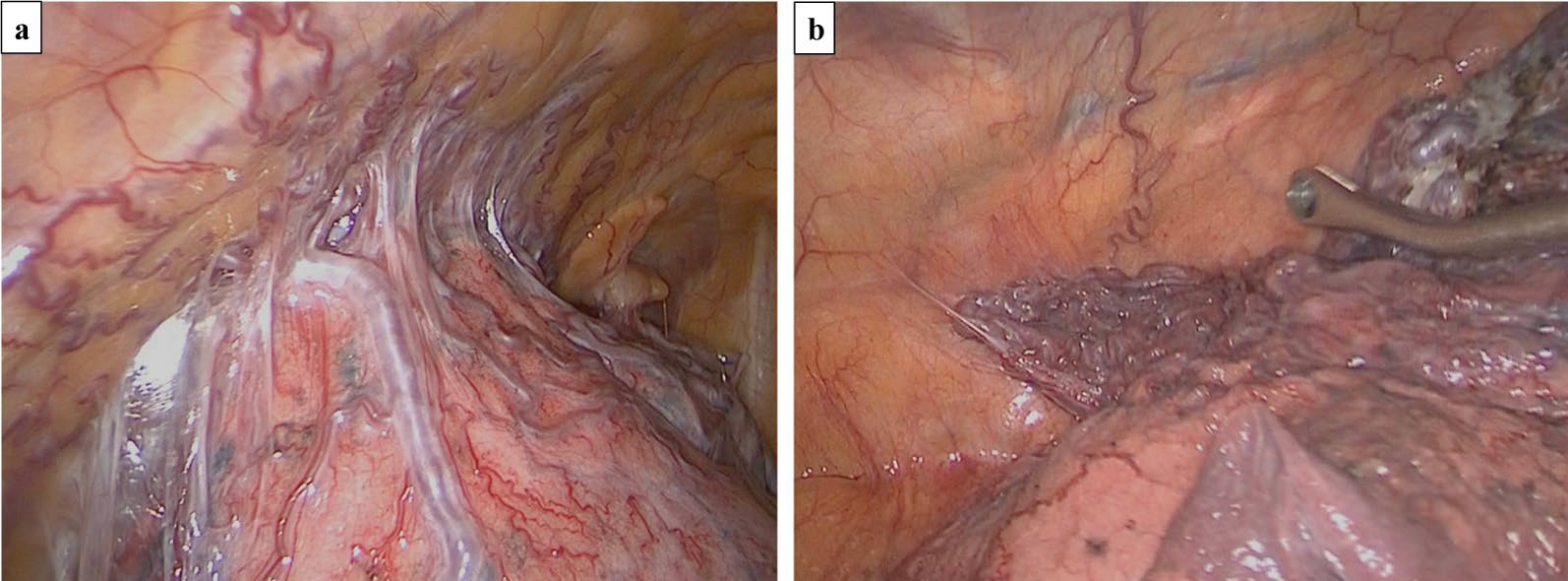
**a** The SAPVF was identified on the surface of the upper lobe, and huge bullae were found in the apex of the upper lobe (**a**). **b** Another SAPVF was also found in the lingular segment with many abnormal vessels from the chest wall (**b**)

The patient was placed in the right lateral position. An Airseal/12-mm port was inserted through the 7th intercostal space (ICS) in the anterior axillary line, and two 12-mm thoracic ports were inserted in the anterior and posterior axillary line through the 4th ICS. The SAPVF seen on the surface of the upper lobe was adherent to the chest wall, with many aberrant blood vessels directed toward the SAPVF (Fig. 3a). Huge bullae were also found at the apex of the upper lobe. Aberrant vessels were separated from the chest wall using a vessel sealing device. We then performed wedge resection, including the bullae, using autosuture devices. Another SAPVF was also seen in the lingular segment with many abnormal vessels from the chest wall, and wedge resection was performed to remove this lesion (Fig. 3b).

The patient’s postoperative course was uneventful with resolution of his symptoms. The patient was discharged on postoperative day 9. The patient had an uneventful postoperative course and his dyspnea improved slightly. The patient remains alive without any symptoms after 6 months of follow-up.

## Discussion

SAPVF is a vascular anomaly characterized by the penetration of systemic chest wall arteries into the lung parenchyma and was first reported by Burchell and Clagett in 1947 [3]. SAPVF can be divided into congenital and non-congenital types. Approximately 15% of SAPVF are congenital [1], and non-congenital types develop in patients with postoperative, with trauma, or with various inflammatory or infectious diseases of the lung and pleura such as pneumonia, pneumothorax, or lung abscess [1, 2]. Specifically, most non-congenital types have been reported to be postoperative cases such as coronary artery bypass graft (CABG) or VATS surgery, and non-postoperative cases in adult are rare.

We compiled a list of cases with non-congenital, non-postoperative SAPVF described in the relevant literature [4–7]. The causes of these cases were listed as a complications of Takayasu's disease, trauma, and or various inflammatory or infectious diseases. In the present case, there was no obvious history of surgery, trauma, or various inflammatory or infectious disease. Furthermore, there were no serological or pathological findings suggesting the presence of fungal infection. The history of heavy smoking, and the presence of giant bullae and diffuse emphysematous changes suggested the presence of chronic inflammation in the thoracic cavity, which was considered to be the cause of SAPVFs.

The SAPVF has few symptoms and is often discovered by chance during routine physical examination. Patients with symptoms present with hemoptysis, dyspnea, pulmonary hypertension, and chronic chest pain. [2, 8]. The natural outcome of SAPVF is not yet well known. Described complications include hemoptysis or congestive heart failure [9]. Therefore, several authors said that intervention is recommended because of the future risk of endocarditis, angina pectoris, and congestive heart failure [9]. Contrast-enhanced CT should always be performed for SAPVF, as it plays an important role in detecting the causes of SAPVF and related complications. Angiography is considered essential in the diagnosis of SAPVF. It makes the full anatomic structure of the lesion more obvious and can clarify the extent and location of the lesion.

SAPVF can be treated by embolization or surgical resection. Recently, embolization has become the preferred method because it causes minor trauma, minimal loss of lung parenchyma, and does not require general anesthesia [9]. Embolization is thought to be more effective in patients with a single or few vessels than in those with many abnormal vessels. In patients, as in our case, who have many abnormal vessels, surgery is the treatment of choice to cure and prevent recurrence of SAPVF. Multiple non-congenital SAPVFs which had no obvious history of surgery, trauma, or various inflammatory or infection disease are very rare, and this case is the first report to describe the surgical treatment of multiple SAPVFs.

Surgical treatment includes local excision, segmental resection, lobectomy, ligation, and even pneumonectomy [10]; in recent years, VATS has been actively performed [11]. In the current case, adhesions between the SAPVF and chest wall, which contained many aberrant blood vessels, could be safely separated using a vessel sealing system during VATS. In fact, no complications, such as bleeding, were observed intra- or post-operatively, and the surgery was performed safely. Therefore, surgery should be considered as the first choice for SAPVF that is difficult to treat with embolization.

## Conclusion

We reported a case of multiple SAPVFs that was safely treated with complete wedge resection using VATS. Resection using VATS should be the first choice of treatment for patients with many abnormal vessels or multiple lesions.

## Data Availability

The datasets used and/or analyzed during the current study are available from the corresponding author on reasonable request.

## Abbreviations

SAPVF: Systemic artery to pulmonary vessel fistula
VATS: Video-assisted thoracoscopic surgery
CT: Computed tomography
SpO2: Percutaneous oxygen saturation
ICS: Intercostal space
CABG: Coronary artery bypass graft
